# Inhibitory Effects of β-Caryophyllene on *Helicobacter pylori* Infection In Vitro and In Vivo

**DOI:** 10.3390/ijms21031008

**Published:** 2020-02-03

**Authors:** Hyun Jun Woo, Ji Yeong Yang, Min Ho Lee, Hyun Woo Kim, Hye Jin Kwon, Min Park, Sung-kyu Kim, So-Young Park, Sa-Hyun Kim, Jong-Bae Kim

**Affiliations:** 1 Department of Biomedical Laboratory Science, College of Health Sciences, Yonsei University, Wonju 26493, Korea; taesube@nate.com (H.J.W.); clever1088@nate.com (J.Y.Y.); amaranth1001@nate.com (H.W.K.); haejin5462@naver.com (H.J.K.); 2 Department of Clinical Laboratory Science, College of Medical Sciences, Daegu Haany University, Gyeongsan 38610, Korea; 3 Forensic DNA Division, National Forensic Service, Wonju 26460, Korea; lmh77777@naver.com; 4 Department of Biomedical Laboratory Science, Daekyeung University, Gyeongsan 38547, Korea; pm@tk.ac.kr; 5 SFC BIO Co., Ltd. 1505-1ho, Daerung-town, 25, Gasan digital 1 ro, Geumcheon-gu 08594, Seoul, Korea; skkim@sfcbio.com; 6 College of Pharmacy, Dankook University, 119 Dandae-ro, Cheonan-si, Chungnam 31116, Korea; soypark23@dankook.ac.kr; 7 Department of Clinical Laboratory Science, Semyung University, Jaecheon 27136, Korea

**Keywords:** antimicrobial, β-caryophyllene, *Helicobacter pylori*, Mongolian gerbil, natural compound

## Abstract

The human specific bacterial pathogen *Helicobacter pylori* (*H. pylori*) is associated with severe gastric diseases, including gastric cancer. Recently, the increasing resistance makes the usage of antibiotics less effectively. Therefore, development of a new antimicrobial agent is required to control *H. pylori* infection. In the current study, the inhibitory effect of β-caryophyllene on *H. pylori* growth, as well as the antibacterial therapeutic effect, has been demonstrated. β-caryophyllene inhibited *H. pylori* growth via the downregulation of *dna*E, *dna*N, *hol*B, and *gyr*A and also downregulated virulence factors such as CagA, VacA, and SecA proteins. β-caryophyllene inhibited expression of several T4SS components, so that CagA translocation into *H. pylori*-infected AGS gastric cancer cells was decreased by β-caryophyllene treatment. β-caryophyllene also inhibited VacA entry through the downregulation of T5_a_SS. After β-caryophyllene administration on Mongolian gerbils, the immunohistochemistry (IHC) and Hematoxylin&Eosin stains showed therapeutic effects in the treated groups. Hematological data, which was consistent with histological data, support the therapeutic effect of β-caryophyllene administration. Such a positive effect of β-caryophyllene on *H. pylori* infection potently substantiates the natural compound as being capable of being used as a new antimicrobial agent or functional health food to help patients who are suffering from gastroduodenal diseases due to *H. pylori* infection.

## 1. Introduction

*Helicobacter pylori* (*H. pylori*) is a Gram-negative, spiral-shaped, microaerophilic bacterium that selectively colonizes in human gastric mucosa [1,2]. Compared with uninfected individuals, *H. pylori* infected individuals have a 2−8-fold increased risk of developing gastric cancer [3]. For these reasons, *H. pylori* has been classified as class I carcinogen by the World Health Organization (WHO) [4]. *H. pylori*-mediated gastric diseases are mostly due to the effect of its virulence factors. Thus, understanding the biological characteristics and mechanisms of their virulence factors might offer a more comprehensive understanding into the pathogenesis of *H. pylori* infection.

Cytotoxin-associated protein A (CagA) is a highly immunogenic protein that is encoded by the *cag* pathogenicity island (PAI). The *cag*PAI genes also encode components of a type IV secretion system (T4SS), which injects CagA protein into host cells [5]. The T4SS apparatus commonly consists of 11 VirB proteins, encoded by the *vir*B1-B11 genes and VirD4 protein [6,7]. Once translocated, CagA dysregulates the homeostatic signal transduction of the gastric epithelial cells, leading the loss of cell polarity, apoptosis, and proliferation, which are involved in chronic inflammation and gastric cancer [8,9]. 

Vacuolating cytotoxin A (VacA) protein is a known pore-forming secreted toxin found in almost all *H. pylori* strains [10]. VacA secretion is regulated by the type V_a_ secretion system (T5_a_SS) and depends on the Sec machinery for transport through the inner membrane [11]. Secretion system subunit protein A (SecA) is an intracellular ATPase that provides the necessary energy for the translocation of *H. pylori* proteins out of the bacterial plasma membrane [11,12,13]. Following translocation into the host cells, VacA leads multiple cellular alterations, including vacuolation and cell death [14]. 

DNA replication is the biological process of copying the DNA in all living organisms. Initiation of DNA replication occurs after the binding of the initiator protein DnaA to the AT-rich regions on *ori*C. DnaA then forms a complex with other nucleoproteins (DnaBC) and ATPs to make the pre-Replicative Complex [15]. DnaB is DNA helicase that progressively unwinds the double stranded DNA in the 5′-3′ direction [16]. *H. pylori* possesses six DNA polymerase III holoenzyme genes. These include two genes for replicase (*dna*E and *dna*Q), one for clamp (*dna*N) and three for clamp loader (*dna*X, *hol*A and *hol*B) [15]. The termination of DNA replication takes place when the helicase DnaB on the leading strands reach a Tus protein [17].

β-caryophyllene is a volatile bicyclic sesquiterpene compound that exists as a mixture of mainly β-caryophyllene and small amounts of α-humulene [18]. It is easily found in the essential oils of many edible plants such as cloves (*Eugenia caryophyllata*) [19], oregano (*Origanum vulgare*), and cinnamon (*Cinnamomum* spp.) [18,20,21]. Recent approval of β-caryophyllene as a food additive and flavoring agent by the Food and Drug Administration in the USA (FDA) and the European Food Safety Authority (EFSA) generated interest among the scientific community to explore its additional therapeutic benefits [22]. Numerous reports showed inhibitory effects of β-caryophyllene against bacteria, virus, and fungi [23,24,25,26]. 

Recently, natural compounds derived from medicinal plants seem to be an important source of antimicrobial agents. Due to the increasing resistance and the emergence of adverse effects, the usage of antibiotics and antibacterial therapeutics is becoming less effective. Therefore, inhibitory effect of β-caryophyllene on *H. pylori* growth and its inhibitory mechanisms were investigated in this study. Furthermore, we aimed to discover a new antimicrobial agent to eradicate *H. pylori,* or to identify a new functional health food that can reduce the virulence of *H. pylori* in infected gastric cells in vitro and in vivo.

## 2. Results and Discussion

### 2.1. Inhibitory Effect of β-Caryophyllene on the Growth and Expression of Replication Genes of H. pylori

**Figure 1:**
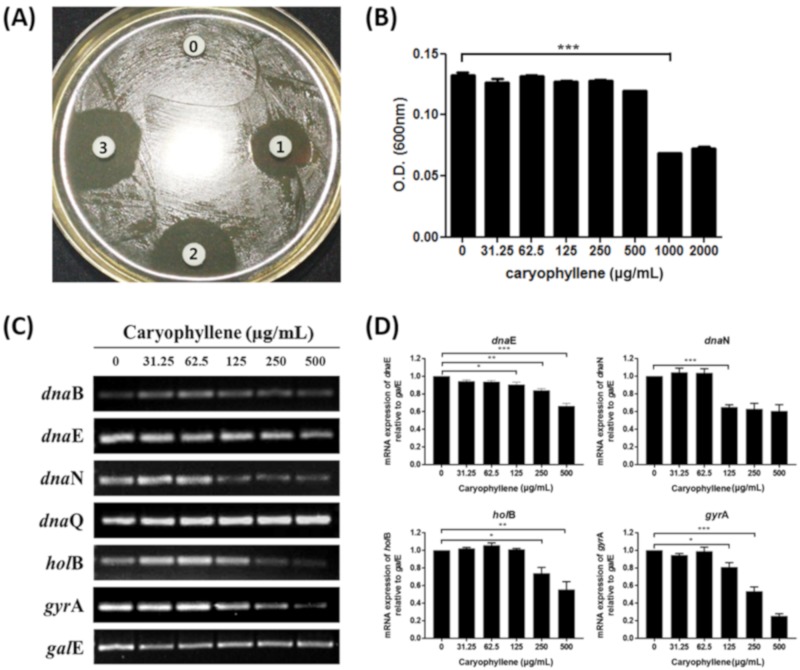
Anti-bacterial activity of β-caryophyllene against *H. pylori* and downregulation of replication-related genes of *H. pylori*. (**A**) Growth inhibitory activity of β-caryophyllene against *H. pylori* was confirmed by a disc diffusion test. Disk 0, control; disk 1, β-caryophyllene 10 μg; disk 2, β-caryophyllene 50 μg; and disk 3, β-caryophyllene 100 μg. (**B**) Minimal inhibitory concentration of β-caryophyllene against *H. pylori* was determined by the broth dilution method. Results from triplicate experiments were analyzed by Student’s *t*-test (** *p* < 0.001). (**C**) The mRNA level expression levels of DNA replication machineries. Constitutively expressed *gal*E was used as an internal control. (**D**) Density of the bands were illustrated as a graph and the results from triplicate experiments were analyzed by Student’s *t*-test (* *p* < 0.05, ** *p* < 0.01 and *** *p* < 0.001).

The inhibitory effect of β-caryophyllene on *H. pylori* was previously evaluated by a screening test using the disc diffusion assay. Clear inhibitory zones were observed around 10, 50, and 100 μg discs and the diameters of the inhibitory zones were 17, 21, and 23 mm, respectively (Figure 1A). By the broth dilution test, the minimum inhibitory concentration (MIC)MIC of β-caryophyllene against *H. pylori* (ATCC 49503) was determined to be 1000 μg/mL (Figure 1B). Only one strain was used to confirm the inhibitory effect of β-caryophyllene, and therefore, it will be necessary to it apply to other reference strains or clinical isolates.

To elucidate how β-caryophyllene inhibits the growth of *H. pylori*, expressions of replication genes of *H. pylori* were evaluated by reverse transcriptase-polymerase chain reaction (RT-PCR). β-caryophyllene treatment decreased the mRNA expression levels of *dna*E, *dna*N, *hol*B, and *gyr*A genes (Figure 1C,D). DnaE is the catalytic α subunit of DNA polymerase III. It has been reported that the mutant *dna*N was unable to support *E. coli* growth [27]. Song *et al.* have shown that *E. coli* strains bearing chromosomal knockout of *hol*B gene was not viable [28]. These studies demonstrated that the DnaE, DnaN, and HolB are necessary for cell growth, and thus they are indispensable. DNA gyrase is pivotal for the process of bacterial replication, so that it has received the most attention in developing antibiotics such as novobiocin (the ATP site inhibitor) and fluoroquinolone (the catalytic site inhibitor) [29,30]. Therefore, interruption of bacterial replication via downregulation of *dna*E, *dna*N, *hol*B, and *gyr*A genes by β-caryophyllene may explain the inhibitory mechanism of β-caryophyllene against *H. pylori* growth. 

### 2.2. Suppression of H. pylori-Induced Apoptosis in Gastric Epithelial Cells by β-Caryophyllene

**Figure 2:**
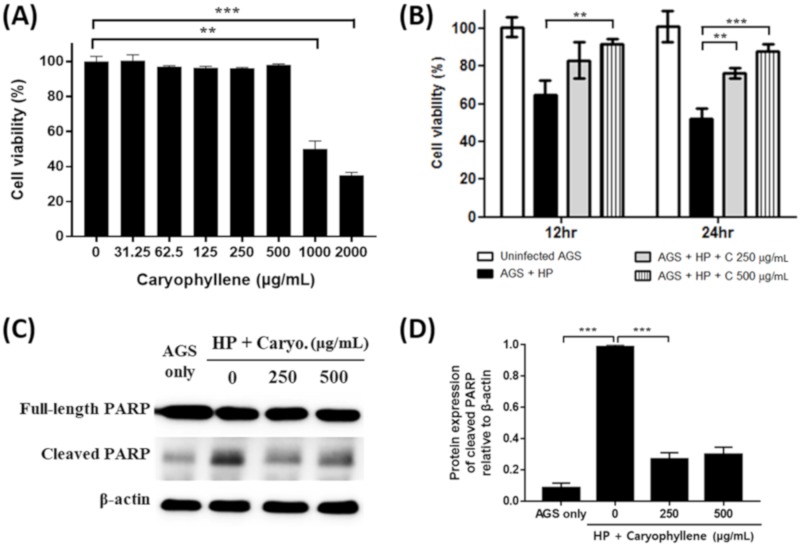
Inhibitory effect of β-caryophyllene on *H. pylori*-infected AGS gastric cancer cell death. (**A**) AGS cells were treated with the indicated concentrations of β-caryophyllene for 24 h and cell viability was measured by the water soluble tetrazolium salt (WST) assay. Cell viability of AGS cells was decreased with 1000 μg/mL or higher dose of β-caryophyllene treatment. AGS cells were infected with *H. pylori* (200 MOI) and treated with β-caryophyllene. (**B**) After 12 or 24 h, cell viability was measured the WST assay. (**C**) The cell lysates were assessed by Western blotting to detect a full-length of poly ADP-ribose polymerase (PARP, 116 kDa) and cleaved PARP (89 kDa). β-actin was used as an internal control. (**D**) The density of the bands are illustrated as a graph. Data in the bar graphs are presented as the mean ± SEM. Data were from the three independent experiments and analyzed by unpaired Student’s *t*-test (** *p* < 0.01 and *** *p* < 0.001).

Infection of *H. pylori* results in deleterious effects on gastric epithelial cells such as the induction of apoptosis, which is closely related to gastric cancer development [31]. Thus, it was evaluated whether β-caryophyllene can alleviate the deleterious effects of *H. pylori* infection on gastric epithelial cells. β-caryophyllene showed no cytotoxic effect on AGS gastric cancer cells without *H. pylori* infection up to 500 μg/mL (Figure 2A) and *H. pylori* infection (200 MOI) reduced cell viability of AGS cells to 51.8% in 24 h (Figure 2B). However, the reduced cell viability was alleviated up to 87.6% by 500 μg/mL β-caryophyllene treatment (Figure 2B).

**Figure 3:**
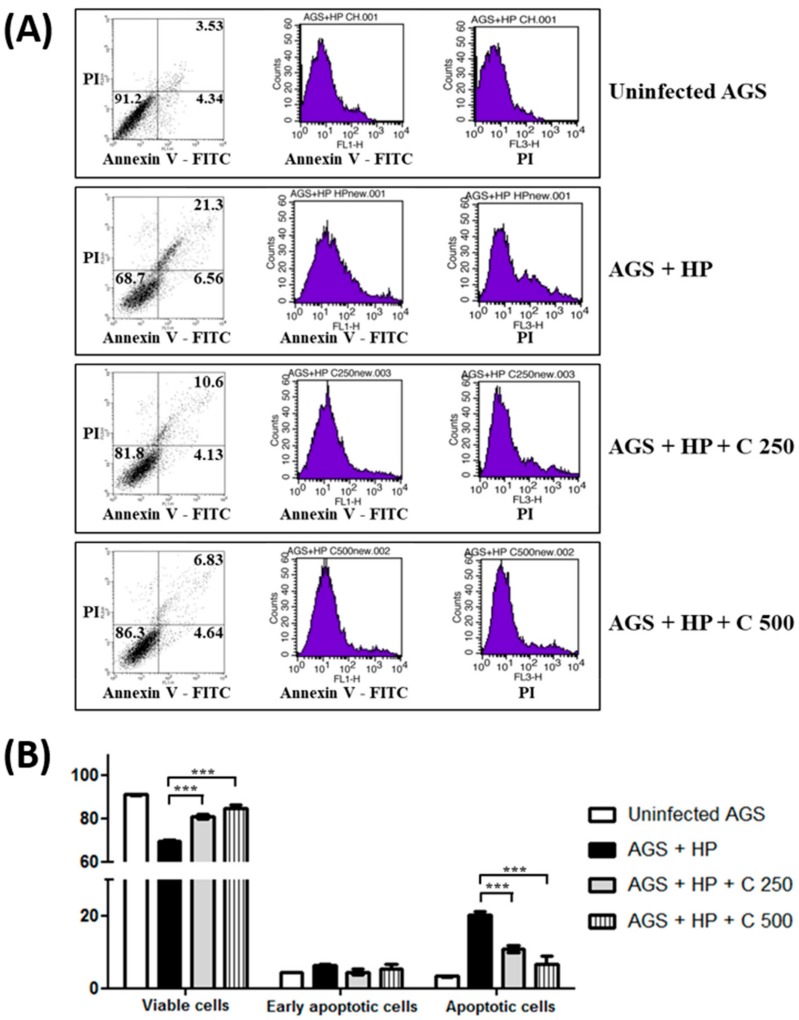
Effect of β-caryophyllene on apoptosis of AGS cell infected with *H. pylori*. AGS cells were infected with *H. pylori* (200 MOI) and treated with indicated dose of β-caryophyllene (250 and 500 μg/mL) for 24 h. After incubation, the cells were stained with annexin V-FITC and PI and subjected to flow cytometry. β-caryophyllene alleviated apoptosis of AGS cells induced by *H. pylori* infection. (**A**) Stained cells were analyzed and illustrated on the quadrant by CellQuestPro software. (**B**) The percentage of cells in apoptosis was analyzed and illustrated as a graph. Results from triplicate experiments were analyzed by Student’s *t*-test (*** *p* < 0.001).

In the Western blot analysis, poly ADP-ribose polymerase (PARP) was cleaved in AGS cells by *H. pylori* infection, indicating the induction of apoptosis. The *H. pylori*-induced PARP cleavage was inhibited by β-caryophyllene treatment (Figure 2C,D). Furthermore, annexin V-FITC stain result also showed that apoptosis induced by *H. pylori* infection was alleviated in AGS cells by β-caryophyllene treatment (Figure 3A,B). These results collectively suggest that β-caryophyllene inhibited apoptosis and alleviated cell death in AGS cells infected with *H. pylori*.

### 2.3. Inhibitory Effects of β-Caryophyllene on the Translocation of Bacterial CagA and VacA Proteins

**Figure 4:**
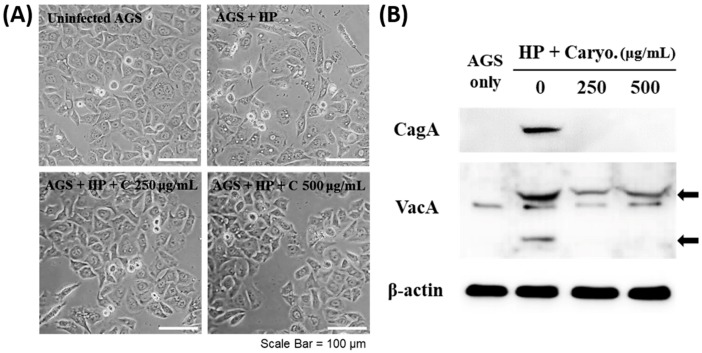
Inhibitory effect of β-caryophyllene on CagA and VacA translocation into AGS cells by *H. pylori*. AGS cells were infected with *H. pylori* (200 MOI) and treated with indicated dose of β-caryophyllene (250 and 500 μg/mL) for 24 h. After incubation, (**A**) morphological changes were observed with an inverted microscope (×200). (**B**) The cell lysates were assessed by Western blotting to detect translocated CagA and VacA proteins to AGS cells.

Cytoskeletal rearrangement and resultant morphological change so-called hummingbird phenotype is a noted outcome appearing after injection of CagA protein into AGS cells and the accumulation of cytoplasmic vesicles (vacuolation) is induced by VacA translocation. β-caryophyllene treatment diminished the hummingbird phenotype and vacuolation on *H. pylori*-infected AGS cells dose dependently (Figure 4A) and also inhibited CagA and VacA protein translocation into the *H. pylori*-infected AGS cells (Figure 4B). 

**Figure 5:**
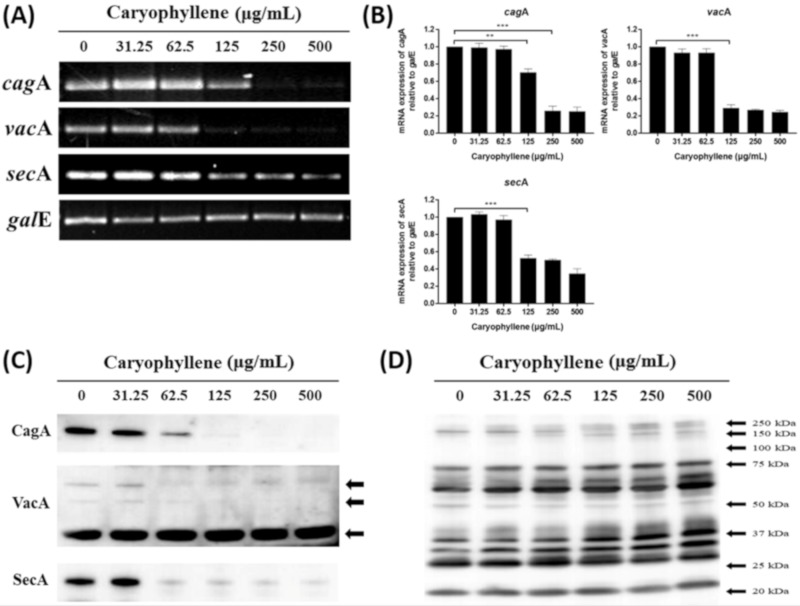
Expression of virulence factors in *H. pylori* treated with β-caryophyllene. *H. pylori* was treated with indicated concentrations of β-caryophyllene in Mueller-Hinton broth for 72 h. (**A**) The RNA was extracted and subjected to Rt-PCR to detect the expression levels of virulence factors. Constitutively expressed gale was used as an internal control. (**B**) Density of the bands were illustrated as a graph and the results from triplicate experiments were analyzed by Student’s *t*-test (** *p* < 0.01 and *** *p* < 0.001). (**C**) The bacterial lysates were assessed by Western blotting to detect CagA, VacA, and SecA protein. (**D**) Rabbit anti-*H. pylori* polyclonal antibody was used as an internal control.

**Figure 6:**
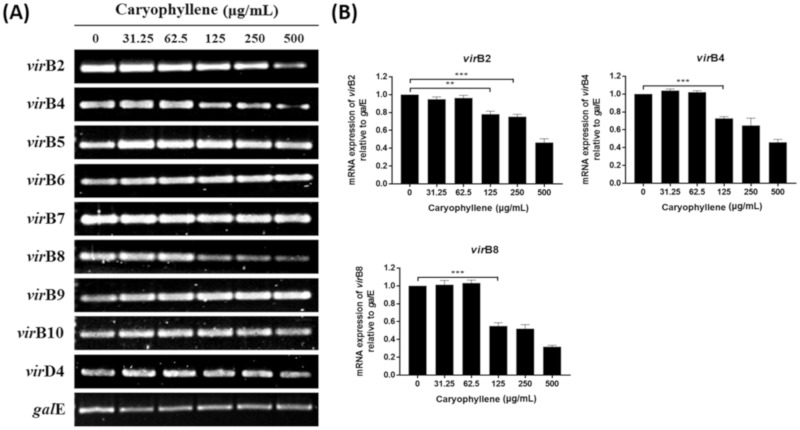
Inhibitory effect of β-caryophyllene on the CagA translocation through T4SS. (**A**) *H. pylori* was treated with indicated concentrations of β-caryophyllene and the RNA was extracted. The collected RNA was subjected to reverse transcriptase-polymerase chain reaction (RT-PCR) to detect the expression of T4SS components. The *gal*E was used as an internal control. (**B**) The density of the bands were illustrated as a graph, and the results from triplicate experiments were analyzed by Student’s *t*-test (** *p* < 0.01 and *** *p* < 0.001).

To elucidate the reasons why the translocation of CagA and VacA proteins to AGS cells was inhibited by β-caryophyllene, we investigated the effect of β-caryophyllene on *H. pylori* directly on the mRNA and protein expressions of CagA and VacA. Both mRNA and protein levels of CagA, VacA and SecA in *H. pylori* were reduced by β-caryophyllene treatment (Figure 5). Besides, the mRNA expression levels of *vir*B2, *vir*B4, *vir*B8, and T4SS components in *H. pylori,* were reduced in a β-caryophyllene dose-dependent manner (Figure 6). These results suggest that inhibition of CagA and T4SS expressions by β-caryophyllene might be, in part, associated with the decreased translocation of CagA to AGS cells and downregulated SecA, as well as VacA might be induced VacA translocation to AGS cells. Collectively, these in vitro data provides evidence supporting that β-caryophyllene may helpful in attenuating the deleterious effects such as hummingbird phenotype, vacuolation, and apoptotic cell death induced by *H. pylori* infection. 

### 2.4. Therapeutic Efefcts of β-Caryophyllene on Mongolian Gerbils Infected With H. pylori

The in vivo experiments in this study demonstrated the therapeutic effects of β-caryophyllene. Mongolian gerbils in each group were sacrificed at 0 week, 6 weeks, and 12 weeks after beginning the β-caryophyllene treatment. RNA was extracted from stomach of the Mongolian gerbils, and then RT-PCR targeting *H. pylori*’s 16S rRNA was performed to evaluate the presence of living *H. pylori*. The result at 0 week showed successful colonization of *H. pylori* in all the infected groups. The presence of *H. pylori* was detected in all the *H. pylori* infected Mongolian gerbils without β-caryophyllene treatment, and the infection lasted until 12 weeks. However, *H. pylori* was not detected in the groups treated with β-caryophyllene since 6 weeks, indicating the successful eradication of *H. pylori* by β-caryophyllene (Figure S2).

**Figure 7:**
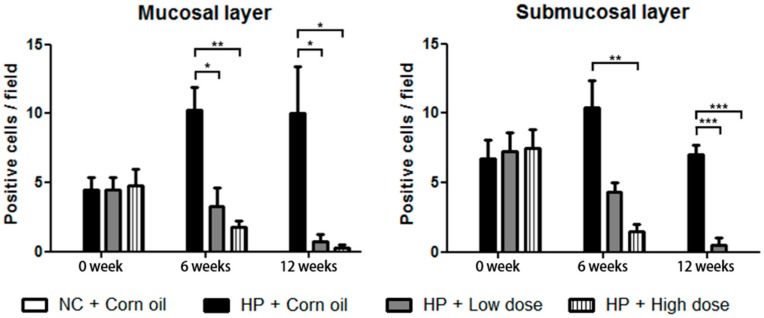
Immunohistochemistry of gastric mucosal and submucosal layer in *H. pylori*-infected Mongolian gerbils. Statistical analysis of data obtained from photomicrograph images (Figures S3 and S4). *H. pylori*-positive cells were identified by counting the number of cells staining intensely in five fields from each sample and 2−3 gastric tissues were assessed for each group. Data in the bar graphs are presented as the mean ± SEM. Results were analyzed by Student’s *t*-test (** *p* < 0.01 and *** *p* < 0.001).

Furthermore, gastric tissue sections of each group were prepared and presence of *H. pylori* was also investigated by using the IHC stain. Anti-*H. pylori* antibody was used to detect the presence of *H. pylori*. There was no positive signal observed in the uninfected gerbil group (normal control group). In contrast, positive signals for anti-*H. pylori* antibody were observed abundantly in gastric mucosal and the submucosal layer of β-caryophyllene-untreated group (*H. pylori* control group), which indicated a marked *H. pylori* infection in the gastric epithelium of the gerbils. *H. pylori* were also detected in the β-caryophyllene-treated groups at 0 week, but the positive signals were decreased over time after β-caryophyllene treatment, suggesting that both low doses (100 μg/g) and high doses (500 μg/g) of β-caryophyllene significantly reduced the degree of infection by *H. pylori* (Figure 7, Figures S3 and S4).

### 2.5. Inhibitory Effects of β-Caryophyllene on the H. pylori-Induced Inflammation

**Figure 8:**
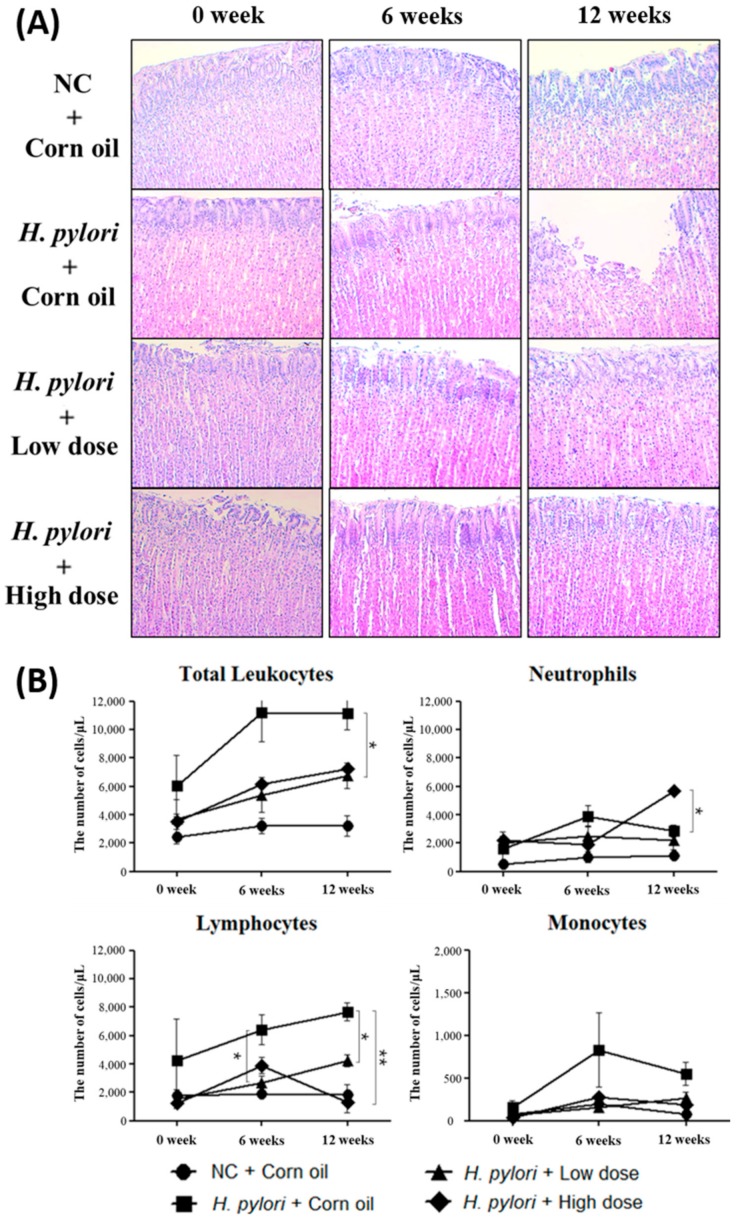
H&E staining of gastric mucosal layer and blood leukocyte count in *H. pylori*-infected Mongolian gerbils. (**A**) The stomach tissues from *H. pylori*-infected Mongolian gerbils were collected for H&E stain. Photomicrograph shows gastric mucosal layer of *H. pylori*-infected Mongolian gerbils (×200). (**B**) Blood was taken from *H. pylori*-infected Mongolian gerbils at the specified time after β-caryophyllene administration (0, 6, and 12 weeks) and subjected to blood cell counting. Data in the bar graphs are presented as mean ± SEM. The results were significant (* *p* < 0.05 and ** *p* < 0.01), as compared with the *H. pylori* control. Circle, NC + corn oil group; square, *H. pylori* + corn oil group; triangle, *H. pylori* + low dose group; diamond, *H. pylori* + high dose group.

H&E staining data showed that β-caryophyllene treatment diminishes inflammation in *H. pylori*-infected stomach tissues (Figure 8A). The reason why inflammatory signs are decreased in β-caryophyllene-treated groups is due to the fact that colonized *H. pylori* might be eradicated by β-caryophyllene administration. In a long-term in vivo study, Wiedemann et al. reported that early inflammation was observed at 8 weeks and precancerous gastric changes were developed at late time points (32 or 64 weeks) [32]. According to these findings, it might be able to detect more severe inflammatory signs and precancerous changes from the *H. pylori* control group, if the infection was maintained over 32 or 64 weeks. 

In the hematological study, the numbers of total leukocytes in *H. pylori* control group were vertically increased at 6 weeks and maintained highly at 12 weeks. The numbers of total leukocytes in β-caryophyllene-treated groups, however, were gradually increased after β-caryophyllene administration (Figure 8B). Detailed comparison of hematological changes between β-caryophyllene-untreated group and β-caryophyllene-treated groups also showed that the treatment of β-caryophyllene alleviates *H. pylori* infection. As neutrophils actively resist bacterial infection, the increased-neutrophil count is commonly found in bacterial infection. However, elevated numbers of neutrophils were relieved by β-caryophyllene administration. This finding implies that bacterial infection is dwindled or bacterial pathogenesis is hindered. In addition to neutrophils, the increased number of lymphocytes and monocytes were lessened at 6 and 12 weeks after β-caryophyllene administration. Contrary to human leukocytes, the composition ratio of lymphocytes in total leukocytes is over 60% in Mongolian gerbils [33,34]. Resulting from the ratio, total changes of leukocytes may be mostly affected by the changes of lymphocytes in Mongolian gerbils. These data correspond with preceded histological data and this certainly supports the therapeutic effect of β-caryophyllene on *H. pylori* infection. Meanwhile, there may be controversy concerning the neutrophil count data because of the sudden fluctuation in the high dose group at 12 weeks. It is speculated that daily medication with a high dose of β-caryophyllene may lead to the chemical toxicity.

The development of new antimicrobial agents with fewer disadvantages is necessary for the eradication of *H. pylori*. β-caryophyllene, which is one of the natural compounds that is easily found in the essential oils of many plants [19,20,21]. According to several reports, β-caryophyllene has antibacterial effect on cariogenic bacteria and food-spoilage bacteria [23,24,25]. This study demonstrated the inhibitory effect of β-caryophyllene on *H. pylori* growth and the protective effect against *H. pylori* infection, as well as an antibacterial therapeutic effect. The aim of this study was to discover the new antimicrobial agent from nature that may inhibit the *H. pylori* infection; thus, this study aimed to investigate the effect of β-caryophyllene as one of the natural compounds that possess multiple pharmacological properties [18]. Further studies are required to fully elucidate about the anti-inflammatory and anti-apoptotic mechanism of β-caryophyllene against *H. pylori*. A previous study from Tambe et al. suggested that β-caryophyllene has a gastric cytoprotective effect [35]. Thus, it would be intriguing to study the anti-inflammatory and anti-apoptotic mechanism of β-caryophyllene during *H. pylori* infection. Along with the mechanisms studied, the oral LD_50_ of β-caryophyllene in Mongolian gerbil seems to be necessary to establish. 

## 3. Materials and Methods

### 3.1. Bacterial Culture and Determination of Antibacterial Activity

*H. pylori* ATCC 49503 strain (East-asian type: CagA^+^/VacA^+^, American Type Culture Collection, Manassas, VA, USA) was grown on Brucella agar plates (Becton-Dickinson, Braintree, MA, USA), supplemented with 10% bovine serum (BRL Life Technologies, Grand Island, NY, USA) at 37 °C for 72 h under a humidified atmosphere with 5% CO_2_. For disc diffusion method, the number of bacteria in the *H. pylori* suspension was adjusted to a McFarland scale 2 (6 × 10^8^ cells/mL) on Mueller-Hinton agar (Becton-Dickinson), supplemented with 10% bovine serum and then incubated for 72 h. To determine the minimum inhibitory concentration (MIC) of β-caryophyllene against *H. pylori*, the number of bacterial particles in the *H. pylori* suspension was set to McFarland scale 0.5 (1.5 × 10^8^ cells/mL). Various concentrations of β-caryophyllene (7.81–4000 μg/mL) were treated and the bacteria were incubated for 72 h and final optical density (600 nm) of the bacterial suspension was measured by using NanoQuant spectrophotometer (infinite M200, TECAN, Männedorf, Switzerland). For normal control, the same volume of ethanol was administrated to culture media. β-caryophyllene was provided by SFC BIO Co., Ltd. Seoul, in republic of Korea.

### 3.2. Mammalian Cell Culture

AGS gastric adenocarcinoma cells (ATCC CRL-1739) were cultured in DMEM medium (BRL Life Technologies), supplemented with 10% fetal bovine serum (BRL Life Technologies) and streptomycin-penicillin (100 μg/mL and 100 IU/mL, BRL Life Technologies). Cells were infected with *H. pylori* as a concentration of 200 multiplicity of infection (MOI) without the addition of antimicrobial agents in media and then treated with β-caryophyllene (250 and 500 μg/mL). For normal control, the equivalent amount of ethanol was administrated to culture media. 

### 3.3. Reverse Transcriptase-Polymerase Chain Reaction (RT-PCR)

*H. pylori* ATCC 49503 strain was grown in Mueller-Hinton broth (Becton-Dickinson) for 72 h. Cultured *H. pylori* was washed twice with phosphate-buffered saline (PBS) and total RNA was extracted using Trizol reagent (Invitrogen, Carlsbad, CA, USA), as described in the manufacturer’s instructions. cDNA was synthesized by reverse transcription with random hexamer (Invitrogen) and Moloney murine leukemia virus-reverse transcriptase (MMLV-RT, Invitrogen). Subsequent PCR amplification was performed in a thermocycler, using specific primers. 

### 3.4. Western Blotting

Bacteria and AGS cells were lysed with radio immunoprecipitation assay (RIPA) lysis buffer (Millipore, Billerica, MA, USA) containing a protease inhibitor cocktail (Calbiochem, San Diego, CA, USA) and protein concentrations were determined based on Lowry method. Antibodies to detect CagA, VacA, and β-actin were purchased from Santa Cruz Biotechnology (Dallas, TX, USA) and polyclonal antibody against whole *H. pylori* (ATCC 49503) and SecA were produced, as previously described [13,36]. Antibodies to detect PARP were purchased from Cell Signaling Technology (Danvers, MA, USA). Anti-*H. pylori* polyclonal antibody and β-actin were used as an internal control for bacteria and mammalian cell proteins.

### 3.5. WST Cell Viability Assay Using EZ-Cytox

AGS cells (1 × 10^4^ per well) were plated in 96-well plates. After 24 h, cells were treated with various concentrations of β-caryophyllene. The cells were then incubated for 24 h and subjected to water soluble tetrazolium salt (WST) assay by using EZ-Cytox cell viability assay kit according to manufacturer’s instruction. Ten μL of WST solutions were added to the cultured media and were incubated for 2 h in the CO_2_ incubator. The absorbance was measured at 450 nm by a spectrophotometer.

### 3.6. Annexin V and PI Staining

Annexin V and PI staining was performed by using Annexin V-FITC Apoptosis Detection Kit I (Becton-Dickinson) according to the manufacturer’s instruction. The cultured cells were detached with 0.25% trypsin-EDTA, washed twice with cold PBS, and centrifuged at 3000 rpm for 5 min. The cells were resuspended in 500 μL of 1X binding buffer at a concentration of 5 × 10^5^ cells/mL and 5 μL of Annexin V-FITC and 5 μL of PI were added to the cell suspension. The mixture was incubated for 10 min at 37 °C in the dark and analyzed by FACS Caliber flow cytometry (Becton-Dickinson).

### 3.7. Animal and Experimental Design

Inbred specific pathogen free (SPF) 5 week-old, male and female Mongolian gerbils for mating were purchased from Central Lab Animals, South Korea. Gerbils used in this study were obtained from 10 breeding cages bred in-house. Animals were divided into four groups. “Normal control group” comprised of 7 gerbils that were inoculated with corn oil. “*H. pylori* control group” comprised of 8 gerbils that were inoculated orogastrically three-times with *H. pylori* (1 × 10^9^ cells) and were given no further treatment. Group, “High dose” and group, “Low dose” were inoculated with *H. pylori* (1 × 10^9^ cells) and the administration of β-caryophyllene was orogastrically administrated every day for 12 weeks. β-caryophyllene was diluted with sterile corn oil at two concentrations. Five hundred μg/g was prepared for high dose group, and one hundred μg/g was prepared for low dose group (Figure S1). All gerbils were sacrificed using CO_2_ euthanasia at different times post-administration (0, 6, 12 weeks). The stomach was excised, opened along the greater curvature. One half was used for a culture study (reisolation) and the extraction of RNA, while the other was used for immunohistochemical and histopathological analyses. The blood samples were taken from all sacrificed gerbils for hematological examination. 

### 3.8. Assessment of Histopathology

*H. pylori* were detected in infected gastric tissues by immunohistochemistry (IHC) using a rabbit anti-*H. pylori* antibody (Abcam, Milton, Cambridge, UK). Positive-staining cells were visualized with diaminobenzidine (DAB) (Vectastain Elite ABC Kit for rabbit; Vector Laboratories, Burlingame, CA, USA) and morphometrically analyzed with Leica DM 2500 microscopy and Leica Application Suite software (version 4.4; Leica microsystems, Heerbrugg, Switzerland). The evaluation of *H. pylori*-positive cells as marker of the infection was performed by counting the *H. pylori*-positive cells distributed in the non-infected control gastric tissue. Paraffin embedded longitudinal sections of antrum and corpus were stained with hematoxylin and eosin (H&E) and evaluated. It was graded for gastritis and mucosal changes and analyzed by a double blind test according to the grading scheme for rodents [32,37].

### 3.9. Statistical Analysis

Data in the bar graphs are presented as mean ± standard error of mean (SEM). All the statistical analyses were performed using GraphPad Prism 5.02 software (GraphPad Software, San Diego, CA, USA). All the data were analyzed by unpaired Student’s *t*-test and *p* < 0.05 was considered to be statistically significant (* *p* < 0.05, ** *p* < 0.01 and *** *p* < 0.001).

### 3.10. Ethics Statement

All *in vivo* experiments and procedures were approved by the Institutional Animal Care and Use Committee (IACUC) of Yonsei University Wonju Campus (approval number: YWC-150612-1, 12 June 2015). All works was conducted in compliance with government regulations, including Welfare Measures for Animal Protection of Ministry of Agriculture, Food and Rural Affairs in republic of Korea.

## Supplementary Materials

Supplementary materials can be found at https://www.mdpi.com/1422-0067/21/3/1008/s1. Figure S1. Experimental protocol *in vivo*. Thirty-one gerbils were divided into four groups. “NC + corn oil” were inoculated corn oil without *H. pylori*. “*H. pylori* + corn oil” inoculated with *H. pylori* (1 × 10^9^ cells) 3 times and were given no further treatment. Group, “*H. pylori* + low dose” and group, “*H. pylori* + high dose” were inoculated with *H. pylori* and β-caryophyllene administration was started 2 weeks after the initial inoculation. The treatment was orogastrically performed during 12-week period; 500 μg/g for high dose group, 100 μg/g for low dose group. All gerbils were sacrificed at specified time of administration (0, 6, 12 weeks). Figure S2. Detection of *H. pylori* in stomach of Mongolian gerbils using RT-PCR. The stomach tissues from *H. pylori* infected Mongolian gerbils were collected for RNA extraction then was subjected to RT-PCR. Colonization of *H. pylori* in stomach of Mongolian gerbils was eradicated after β-caryophyllene administration. *H. pylori*-16S rRNA was used to determine the presence of infection. NC: NC + corn oil; HP: *H. pylori* + corn oil; Low: *H. pylori* + low dose group; High: *H. pylori* + high dose group; PC: Positive control. Figure S3. Immunohistochemistry of gastric mucosal layer in *H. pylori*-infected Mongolian gerbils. Gastric tissue sections of each group were prepared and presence of *H. pylori* was also investigated by using IHC stain. Photomicrograph shows gastric mucosal layer of Mongolian gerbils (×200). Arrows or circles indicate the presence of *H. pylori*. Positive signals for anti-*H. pylori* antibody were observed abundantly in gastric mucosal layer of β-caryophyllene-untreated group (*H. pylori* control group) while there was no positive signal observed in the uninfected gerbil group (Normal control group). *H. pylori* were also detected in the β-caryophyllene-treated groups at 0 week, but the positive signals were decreased over times after β-caryophyllene treatment suggesting that both low dose and high dose of β-caryophyllene significantly reduced the degree of infection by *H. pylori*. Figure S4. Immunohistochemistry of gastric submucosal layer in *H. pylori*-infected Mongolian gerbils. Gastric tissue sections of each group were prepared and presence of *H. pylori* was also investigated by using IHC stain. Photomicrograph shows gastric mucosal layer of Mongolian gerbils (×200). Arrows indicate the presence of *H. pylori*. Positive signals for anti-*H. pylori* antibody were observed abundantly in gastric submucosal layer of β-caryophyllene-untreated group (*H. pylori* control group) while there was no positive signal observed in the uninfected gerbil group (Normal control group). *H. pylori* were also detected in the β-caryophyllene-treated groups at 0 week, but the positive signals were decreased over times after β-caryophyllene treatment suggesting that both low dose and high dose of β-caryophyllene significantly reduced the degree of infection by *H. pylori*.
